# Mitochondria at the Crossroads of Survival and Demise

**DOI:** 10.1155/2019/2608187

**Published:** 2019-12-04

**Authors:** Ulrike B. Hendgen-Cotta, Valentina Giorgio, Livia Hool

**Affiliations:** ^1^Department of Cardiology and Vascular Medicine, West German Heart and Vascular Center, University Hospital Essen, Medical Faculty, University of Duisburg-Essen, Germany; ^2^Institute of Neuroscience (CNR) and Department of Biomedical Sciences, University of Padova, Italy; ^3^School of Human Sciences, The University of Western Australia, Crawley, WA, Australia; ^4^Victor Chang Cardiac Research Institute, Darlinghurst, NSW, Australia

Mitochondria are multifunctional organelles, and their structural and functional integrity is fundamental to cell life. In addition to their critical role in the production of ATP via oxidative phosphorylation and biosynthetic intermediates, mitochondria are also a major hub for cellular Ca^2+^ signaling. Moreover, mitochondria can actively or passively drive cellular demise. They can become the major source of reactive oxygen species (ROS) in pathological and physiological processes, and they are highly vulnerable to damage. Mitochondria represent a point of convergence for a variety of upstream cell death stimuli and undergo structural and functional remodeling with subsequent transmission of signals to downstream executioner proteins. The pathways include death stimuli such as dioxygen, metabolic perturbation, deprivation of survival factors, oxidative stress, Ca^2+^ overload, DNA damage, proteotoxic stress, and oncogene activation.

In this special issue, we provide the readership of this journal with a variety of examples of the importance of mitochondria in both physiological and pathological events, the underlying mechanisms, and how mitochondrial dysfunction can be targeted.

In the current issue, several articles focus on mitochondrial dysfunction in different disorders. The article by G. Rigotto and E. Basso provides an excellent review on the role of mitochondrial dysfunction in metabolic disorders such as Alzheimer's disease, diabetes, and obesity. This insight is complemented by the eminent review of M. L.-H. Huang et al. on the role of the antioxidant response in mitochondrial dysfunction in degenerative diseases and the comprehensive overview of S. Li et al. discussing the role of mitochondria-derived damage-associated molecular patterns (mtDAMPs) in sepsis. The role of mitochondrial permeability transition in mitochondrial disorders with particular emphasis on the mitochondrial FoF1-ATP synthases as the fundamental pore in the mitochondrial inner membrane is highlighted in the extensive review by J. Šileikytė and M. Forte. This channel is activated (among other effectors) by the increase in Ca^2+^ concentration in mitochondria. Linked to the Ca^2+^ importance in mitochondria, the original work by V. Granatiero et al. provides the first evidence that the upregulation of the Ca^2+^ mitochondrial uniporter (MCU) in the brain cortex in vivo causes neuronal death. The article by S. Manna et al. summarizes the current knowledge of placental ageing in adverse pregnancy outcomes associated with preeclampsia. L. P. Thompson et al. report that chronic intrauterine hypoxia leads to mitochondrial dysfunction resulting in increased vulnerability to cardiovascular disease in males compared to females, whereas the study by F. L. Sheeran et al. finds upregulation of the mitochondrial pyruvate dehydrogenase (PDH) in end-stage human heart failure, a multienzyme complex at the nexus of glycolytic and Krebs cycles, and affords the severely failing left ventricle crucial capacity to utilize glucose-dependent energy production in the face of dwindling energy options. A case report presented by M. De Luise et al. find that mitochondrial respiration impairment is associated with dysfunctional HIF1*α* by hyperhydroxylation in renal oncocytoma. S. Gonnissen et al. report that high concentrations of low-density lipoprotein (LDL) impair endothelial cell function caused by a decrease in active nitric oxide synthase 3. This leads to dysregulation of mitochondrial transcription and reduced ATP content and migratory capacity.

Another area of research that is presented is the prevention and treatment of mitochondrial dysfunction. T.-K. Lin et al. report bioenergetic therapeutic effects through transfer of healthy mitochondria by Wharton's jelly mesenchymal stem cells to human fibroblasts harboring a mitochondrial DNA defect, whereas Y. Xin et al. demonstrated that in the hypertrophic heart, the inhibition of mitofusin 2 promotes cardiac fibroblast activation. In a human cell line, the biosynthesis of coenzyme Q and ATP production can be recovered by treatment with vanillic acid, an oxidation product of the nontoxic compound vanillin, as shown by M. J. Acosta Lopez et al. In a study by O. Lozano et al., preservation of mitochondrial function and ATP synthesis during oxidative stress can be achieved in rat ventricular myoblast H9C2 cells by the delivery of the encapsulated antioxidant quercetin using nanoparticle technology. The report by Y. Jiao et al. shows that activation of the nuclear factor erythroid 2-related factor 2 (Nrf2), which regulates the gene expression of antioxidative enzymes, protects against mitochondrial damage and dysfunction caused by dental resin monomers. In addition, modulation of histone deacetylase 2 offers a protective effect in acute liver failure by altering mitochondrial apoptosis shown in the study by Y. Wang et al.

Intracellular physiological and pathological signaling pathways are highly orchestrated and integrated at the molecular level with mitochondria. The review by S. Feno et al. highlights the crosstalk between calcium and ROS with focus on the contribution of the mitochondrial calcium uniporter to cardiovascular, skeletal muscle, and neurodegenerative diseases. Moreover, the review by G. Gherardi et al. elucidates the crosstalk between autophagy and mitochondrial Ca^2+^ uptake in the skeletal muscle. J. R. Huertas et al. discuss the benefits of exercise-induced mitochondrial adaptations emphasizing the importance of mitochondrial biogenesis, morphological changes, and increases in respiratory supercomplex formation. A new insight into the lymphocyte granzyme B cell death pathway *via* mitochondrial entry triggering ROS-dependent cell death is provided by D. Martinvalet. The essential role in cellular metabolism and the detrimental consequences of malfunction of mitochondrial F-ATP synthase are discussed at a subcellular level in the extensive review of G. Lippe et al.

In conclusion, the articles presented in this special issue describe the importance of proper mitochondrial function for healthy organ and organism performance and highlight that mitochondrial dysfunction takes the center stage in an ever-increasing number of pathologies. An understanding of the mechanisms leading to pathology is informing the development of therapy for this vital organ.

